# Do people with a different goal-orientation or specific focus make different decisions about colorectal cancer-screening participation?

**DOI:** 10.1371/journal.pone.0213003

**Published:** 2019-02-28

**Authors:** Linda N. Douma, Ellen Uiters, Danielle R. M. Timmermans

**Affiliations:** 1 Department of Public and Occupational Health, Amsterdam Public Health Research Institute, VU University Medical Center, Van der Boechorststraat 7, BT Amsterdam, The Netherlands; 2 National Institute for Public Health and the Environment (RIVM), BA Bilthoven, The Netherlands; Middlesex University, UNITED KINGDOM

## Abstract

**Objective:**

Previous studies have shown that having promotion-oriented goals (e.g. wanting to become healthy) or prevention-oriented goals (e.g. wanting to avoid getting ill) can affect people’s health-related decisions and behaviour by emphasising aspects and information that seem relevant in light of what they want to achieve. However, this issue has not yet been researched regarding colorectal cancer (CRC) screening. With our study, we aimed to examine the relationship between people’s goal-orientation or focus on advantages or disadvantages and their CRC screening participation, as this could provide insights for supporting people in making this complex decision.

**Methods:**

An online survey was carried out among a sample of first-time CRC screening invitees (1282 respondents, response rate 49%). We assessed people’s goal-orientation (i.e. promotion-orientation and prevention-orientation), focus on the advantages or disadvantages of CRC screening, screening participation and main considerations (e.g. cancer is a serious illness) concerning their screening decision.

**Results:**

Generally, CRC screening participants scored higher on both promotion-orientation and prevention-orientation than non-participants. Both CRC screening participation and non-participation were not associated with a dominant goal-orientation. CRC screening participants did show a dominant focus on the advantages of CRC screening. Mediation analysis showed support for our premise that the relationship between people’s goal-orientation or focus on advantages or disadvantages and their screening participation could be (partially) mediated by people’s main considerations concerning CRC screening.

**Conclusion:**

CRC screening participants and non-participants differed in their goal-orientation and focus on advantages or disadvantages. CRC screening participation appears to be associated with a focus on the advantages of CRC screening, which could impede the making of an informed decision. CRC screening non-participation appears not to be associated with any clear goal-orientation or focus, or we have not yet managed to capture this, which could be either beneficial or problematic for making an informed decision.

## Introduction

When making health-related decisions, people have goals they want to achieve, such as getting better or avoiding becoming overweight, which has been shown to influence their decision and decision-making process [1–7]. A well-known theory in this respect is the Regulatory Focus Theory (RFT) [8]. RFT distinguishes between a promotion focus, where people want to achieve a positive end-state (e.g. being healthy), making them focused on desires and possible gains, and a prevention focus, where people want to avoid a negative end-state (e.g. getting ill), making them focused on safety and potential losses [8, 9]. Promotion focus and prevention focus are seen as distinct orientations that people can have towards a decision, but they are not each other’s opposites [8, 10, 11]. This means that people with a strong promotion focus do not necessarily have a weak prevention focus, or vice versa, but a dominant focus can be present [8, 10]. People’s orientation surrounding their goals regarding a decision leads to an emphasis–consciously or unconsciously–on those aspects of the decision that are thought to be relevant in light of what they want to achieve [8, 9, 12]. This affects which information is noticed and how it is interpreted [6, 13–15], with information that matches in wording or framing people’s goal-orientation being noticed and valued more [11, 16, 17]. As people’s goal-orientation affect their information processing, it is also likely that it affects which considerations people have and value most, regarding a decision [14]. Consequently, different behavioural decisions can be made based on the same information, depending on people’s goal-orientation [6, 14, 16, 17].

To our knowledge, people’s goals and orientation surrounding it has not yet been researched regarding preventive colorectal cancer (CRC) screening, or other cancer screenings. Thus far, research on CRC screening participation has often used the Health Belief Model (HBM) [18] or the Theory of Planned Behaviour (TPB) [19] as a framework. Within HBM and TPB, the emphasis lies on assessing people’s beliefs, knowledge, perceived social norm, self-efficacy, and practical barriers, which have all been shown to be associated with people’s decision concerning CRC screening participation [18–21]. In addition, many experts in the field of cancer screening believe that people should make an informed CRC screening decision based on a good understanding and personal weighing of the potential benefits and harms of CRC screening as well as their personal preferences regarding screening [22–25]. However, people’s goal-orientation could affect how they interpret, use and value the information concerning CRC screening [6, 13–15]. In this way, people’s goal-orientation could perhaps be seen as a general orientation affecting the relevance of people’s more specific beliefs and considerations concerning CRC screening (e.g. colon cancer is a serious illness or I feel healthy). Therefore, examining the possible association between people’s goal-orientation and their CRC screening participation could provide useful insights in understandings people’s CRC screening decision-making process. Guided by the synopsis of RFT and its distinction between promotion focus and prevention focus, people with, for example, promotion-oriented goals could decide to participate in CRC screening because they want to achieve or maintain a state of being healthy. On the other hand, they could also decide not to participate in CRC screening because they believe this will contribute to achieving the positive objectives they value. People with prevention-oriented goals could decide to participate in CRC screening because they want to avoid getting cancer, or they could decide not to participate because they want to avoid being exposed to certain risks [8, 11, 15]. As mentioned above, an essential part of the CRC screening decision involves the weighing of potential benefits and harms [24, 26, 27]. It could be argued that promotion-oriented and prevention-oriented goals are related to mainly focusing on the advantages *or* the disadvantages of CRC screening when deciding about participating in it [8], which could also possibly affect how they interpret, use and value the information concerning CRC screening [6, 13–15].

**Fig 1:**
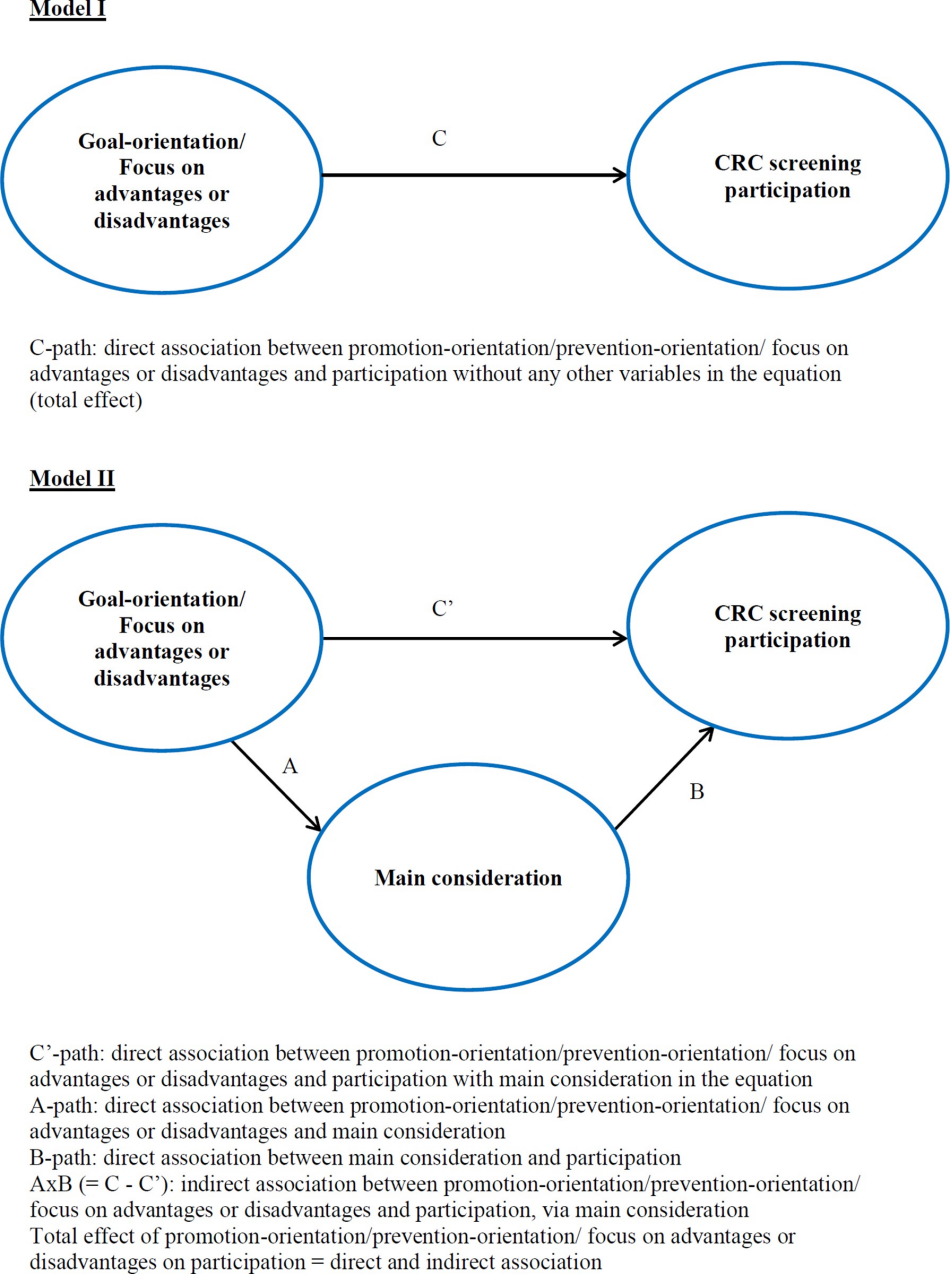
Direct and indirect relationship (mediation) between goal-orientation (promotion- or prevention-oriented) / focus on advantages or disadvantages and main considerations regarding CRC screening and CRC screening participation.

Examining the possible association between people’s goal-orientation, focus on advantages or disadvantages, (main) considerations and decision concerning CRC screening could provide useful insights for developing resources to adequately inform and support people in making their screening decision. In addition, previous studies on CRC screening have shown that participation and beliefs/considerations concerning CRC screening are often associated with people’s sex, age and education [18, 19, 28, 29]. Therefore, it seems relevant to also examine the possible association with these sociodemographic characteristics. More specifically, we aim to answer the following research questions:

1. Is people’s CRC screening participation associated with a specific goal-orientation (i.e. promotion-oriented versus prevention-oriented) or focus on mainly the advantages or disadvantages of CRC screening (Fig 1, Model I)?
  - Is a dominant goal-orientation or focus associated with CRC screening participation or non-participation?
  - Is the relationship between goal-orientation/focus and screening participation mediated by people’s main considerations regarding CRC screening (Fig 1, Model II)?
  - Are people’s goal-orientation/focus and its relationship with CRC screening participation associated with sociodemographic characteristics (sex, age, education)?

## Methods

### Respondents

Participants for our questionnaire were recruited via a national online research panel (I&O Research Panel, www.ioresearch.nl, ISO 26362). Members of this panel are recruited through random sampling, using population records (e.g. municipality records). As an incentive, fifty ten-euro gift cards were raffled among respondents. According to ISO 26362 certificate regulation, people give written consent when signing up to be a member of an online research panel as well as when agreeing to participate in any specific survey sent to them. As possible participants, we were interested in people who were invited for the first time to participate in CRC screening. In the Netherlands, CRC screening is currently gradually being introduced. The following birth years were scheduled to receive an invitation for the first time for CRC screening in 2016: 1941 (age 75), 1945 (age 71), 1953 (age 63), 1955 (age 61), 1957 (age 59). Therefore, 2818 panel members from these birth years were invited via e-mail in March 2017 to complete our online questionnaire. They were told we were interested in hearing people’s views on CRC screening, from both people who decided to participate in CRC screening following their invitation in 2016 and people who decided not to participate. The response rate was 49% (1378 respondents). However, 96 of those respondents were not eligible for our study because they were not invited for CRC screening in 2016, or it was not their first invitation. The Medical Ethics Review Committee of VU University Medical Center (IRB00002991/FWA00017598) has declared that the Medical Research Involving Human Subjects Act (WMO) does not apply to this study and an official approval of this study by their committee is not required.

### Questionnaire

The questions part of this study were part of a larger questionnaire regarding people’s views on CRC screening. A complete version of the questions asked relevant for this study can be found as supplementary material (S1 Appendix), in both the original language (Dutch) and English. Respondents were asked whether they had participated or not in CRC screening before receiving the questions about which considerations had played a role in their decision. This was followed by the questions regarding their focus on the advantages of disadvantages of CRC screening and their goal-orientation.

### Measures

#### Promotion-orientation and prevention-orientation regarding CRC screening participation

In assessing people’s goal-orientation regarding CRC screening participation we were guided by the synopsis of RFT to distinguish between promotion- and prevention-oriented goals. We assessed the extent to which people were promotion-oriented and prevention-oriented by assessing these as separate variables, considering them two distinct orientations [10, 11]. We presented respondents with several statements representing promotion-oriented goals (e.g. maintaining good health) or prevention-oriented goals (e.g. avoiding risks) [8, 30]. Partly different statements were used for CRC screening participants and non-participants as relevant goals in the context of CRC screening participation seem to differ between the two groups (e.g. ‘I want to avoid getting seriously ill’ or ‘I want to avoid unnecessary tests and treatment’). To assess promotion-orientation, CRC screening participants received three statements (final score is the sum-score average; Cronbach’s alpha is .83). As promotion-oriented goals in the context of not participating in CRC screening appear scarce, we realistically could present CRC screening non-participants with only one statement. To assess prevention-orientation, both CRC screening participants and non-participants received three statements (final scores are the sum-score averages; Cronbach’s alpha is .83 and .67, respectively). Respondents were then asked to what extent each statement played a role in their decision concerning CRC screening participation (5-point interval scale with labelled scale points: 1 = did not play a role; 2 = played a small role; 3 = played a role; 4 = played a large role; 5 = played a very large role).

#### Focus on advantages or disadvantages regarding CRC screening participation

In addition to assessing promotion-orientation and prevention-orientation, we assessed people’s focus on advantages or disadvantages regarding CRC screening participation. We asked respondents to choose which of the following two options had played the largest role for them when deciding to participate or not participate in CRC screening (dichotomous variable): 1. I mostly looked at what the advantages of CRC screening could be for me; or 2. I mostly looked at what the disadvantages of CRC screening could be for me.

#### Considerations regarding CRC screening participation

Based on previous research (e.g. [31–34]), we presented respondents with a broad range of possible considerations that may have played a role in their decision concerning CRC screening participation (see S1 Appendix for an overview), and asked them to what extent each consideration played a role (5-point interval scale with labelled scale points: 1 = did not play a role; 2 = played a small role; 3 = played a role; 4 = played a large role; 5 = played a very large role). For descriptive purposes we have categorised these considerations into certain themes: 1. Colon problems and colon cancer (e.g. I feel healthy, colon cancer is a serious illness); 2. CRC screening (e.g. by participating in CRC screening I will avoid serious treatment); 3. Social environment (e.g. I think most people in my environment are positive about CRC screening); 4. Other (e.g. it is difficult to find a suitable time to perform the stool test). However, this categorisation was not used for analysing the results (all considerations were analysed separately).

#### Sociodemographic characteristics

Data on sex, age (birth year) and education (low, intermediate, high; according to the International Standard Classification of Education (ISCED), 2011) were gathered.

### Statistical analysis

#### Construct validity

Confirmatory factor analysis regarding the measure of goal-orientation and the measure of focus on the advantages or disadvantages of CRC screening was performed and confirmed that we are measuring two different factors/constructs (see S3 Appendix). As some correlation between the measures was expected, we used oblique (Direct oblimin) rotation. However, when using orthogonal (Varimax) rotation similar results were found. Reliability analyses (Cronbach’s alpha) were conducted regarding the promotion-orientation scale and prevention-orientation scales and showed acceptable internal consistency.

#### Association between goal-orientation, focus on advantages or disadvantages, main considerations, and participation

Univariate logistic regression analysis and paired t-test were used to examine whether for people’s goal-orientation there was a difference between participants of CRC screening and non-participants. Univariate logistic regression analysis was used to examine whether for people’s focus on the advantages or disadvantages there was a difference between participants of CRC screening and non-participants. Subsequently, we conducted multiple logistic regression analysis to assess people’s main considerations associated with the variable CRC screening participation (participation as dependent variable, with participation in CRC screening scored as 1 and non-participation as 2). All consideration-items were entered as separate variables. Because correlations between the consideration-items are to be expected backward selection was applied (we used the more strict p-value cut-off point of p ≥ .05) [35]. Through backward selection a ‘final model’ is achieved with only considerations significantly associated with the variable CRC screening participation remaining. These considerations were categorised by us as ‘main considerations’. There were no a-priori hypotheses or determinations.

As described in the introduction section, based on existing literature we believe it is possible that people’s goal-orientation or their focus on advantages or disadvantages can affect which considerations people have and value most, and, subsequently, their final decision regarding CRC screening participation [6, 13–15]. To examine this possible relationship mediation analysis was performed, following Baron and Kenny (1986), using univariate and multiple regression analysis [36, 37]. Firstly, we assessed whether a significant association between people’s promotion-orientation and prevention-orientation, each separately, and CRC screening participation existed (C-path in Fig 1, Model I; logistic regression; controlled for confounding by sex, birth year and education). Secondly, we assessed whether a significant association between people’s promotion-orientation/prevention-orientation and each of the main considerations regarding CRC screening separately existed (A-path in Fig 1, Model II; linear regression). Thirdly, we assessed whether a significant association between each of the main considerations separately and CRC screening participation existed, with people’s promotion-orientation/prevention-orientation also included in the model (B-path in Fig 1, Model II; logistic regression). Fourthly, we assessed whether the original association between people’s promotion-orientation/prevention-orientation and CRC screening participation changed after including each of the main considerations separately in the model (i.e. whether this relationship was mediated by people’s main considerations; C’-path in Fig 1, Model II; logistic regression). Lastly, to assess whether these indirect/mediation effect (path A*B/C-C’ in Fig 1, Model II) were significant the Sobel test was performed (which uses beta’s and standard errors). In a similar manner, mediation analysis was also performed to examine whether the relationship between people’s focus on the advantages or disadvantages and CRC screening participation was mediated by people’s main considerations regarding CRC screening. Although at face-value high correlations between the items used to assess goal-orientation/focus on advantages or disadvantages and (some of) the items used to assess people’s main considerations appear possible (thus, possibly prohibiting mediation analysis), correlation analysis does not show extreme high correlations causing potential problems (see S3 Appendix).

#### Association with sociodemographic characteristics

For every analysis performed, we examined possible confounding by sex, birth year and education. When we found no confounding, uncorrected scores are shown. Multiple logistic and linear regression analyses were performed to examine the relationship between CRC screening participation, people’s goal-orientation, people’s focus on advantages or disadvantages, and sex, birth year and education. All analyses were carried out using SPSS 24.0.0.1.

## Results

### Sample characteristics

Our research sample consisted of 1282 respondents (Table 1). More men (60%) than women (40%) participated in our study. Almost half of respondents (48%) were high educated. Most respondents (89%) indicated they had participated in CRC screening. Non-response analysis showed that people were more likely to have participated in our survey when higher educated. No significant differences were found associated with sex or birth year.

**Table 1:** Characteristics research sample.

Variables	N (%)
Total sample	1282 (100)
*Sex*	
Male	773 (60)
Female	509 (40)
*Education*	
Low	258 (20)
Intermediate	404 (32)
High	611 (48)
*Birth year*	
1941	127 (10)
1945	228 (18)
1953	329 (26)
1955	297 (23)
1957	301 (23)
*Screening participation*	
Yes	1142 (89)
No	140 (11)

### People’s goal-orientation, focus on advantages or disadvantages and CRC screening participation

When assessing promotion-orientation and prevention-orientation regarding CRC screening participation, we see that on average CRC screening participants score higher on promotion-orientation and prevention-orientation than CRC screening non-participants (Table 2). The difference between screening participants and non-participants is significant regarding both promotion-orientation (univariate logistic regression analysis: *OR* = .298, *95% CI*: .246 –.361) and prevention-orientation (*OR* = .434, *95% CI*: .365 –.517). Among screening participants the scores on promotion-orientation and prevention-orientation do not significantly differ from each other (paired t-test: *t* (1141) = —.260, *ns*). Among screening non-participants the score on prevention-orientation is significantly higher than the score on promotion-orientation (*t* (139) = - 4.611, *p* < .001). When assessing people’s focus on advantages or disadvantages of CRC screening, we see that most CRC screening participants focus on the advantages, while non-participants are more equally distributed regarding a focus on the advantages or disadvantages. This difference in focus between screening participants and non-participants is significant (*OR* = 83.254, *95% CI*: 46.456–149.232). Regarding all analyses mentioned above, sex, education and birth year were not found to be confounders regarding the relationship between focus and CRC screening participation.

**Table 2:** Goal-orientation and focus on advantages or disadvantages of CRC screening among CRC screening participants and non-participants.

Goal-orientation regarding CRC screening participation	*Total sample*	*CRC screening participants*	*CRC screening non-participants*
	**M (SD)**	**M (SD)**	**M (SD)**
Promotion-orientation ^a^	3.37* (1.03)	3.53 (1.03)	2.05* (1.36)
Prevention-orientation ^a^	3.43* (1.16)	3.54 (1.05)	2.54* (1.14)
**Focus on advantages or disadvantages**	**N (%)**	**N (%)**	**N (%)**
Focus mainly on advantages ^b^	1187* (93)	1125 (98)	62 (44)
Focus mainly on disadvantages ^b^	95* (7)	17 (2)	78 (56)

^a^ Scores range from 1 (low promotion-/prevention-orientation) to 5 (high promotion-/prevention-orientation)

^b^ Dichotomous variable: score 1 = focus mainly on advantages, score 2 = focus mainly on disadvantages

* Significant difference (p < .001) between groups

We also examined whether CRC screening participation, people’s goal-orientation and people’s focus on the advantages or disadvantages of CRC screening were associated with sex, birth year or education (see S4 Appendix). Regarding all variables, no differences were found related to sex. Higher educated people were less likely to have participated in CRC screening compared to lower educated people, and scored lower on both promotion-orientation and prevention-orientation.

### Relationship between people’s goal-orientation, focus on advantages or disadvantages, CRC screening participation and main considerations

The section above describes the direct association between people’s goal-orientation or focus on advantages or disadvantages and their CRC screening participation without any other variables in the equation (‘total effect’; Fig 1, Model I). Subsequently, the aim was to examine whether the relationship between people’s goal-orientation (i.e. their promotion-orientation and prevention-orientation) and CRC screening participation as well as the relationship between people’s focus on advantages or disadvantages and CRC screening participation was mediated by people’s main considerations regarding CRC screening (Fig 1, Model II). S2 Appendix shows the descriptive results of to what extent any of the considerations we presented respondents with played a role in their decision. Generally, it appears that especially for CRC screening participants certain considerations played a role in their decision. As part of the mediation analysis, we first identified the main considerations associated with CRC screening participation using multiple regression analysis (Table 3). A ‘final model’ of eight main considerations significantly associated with CRC screening participation was assessed. Sex, education and birth year were not found to be confounders. Regarding four of these main considerations, people were more likely to participate in CRC screening when these played a larger role in their decision regarding CRC screening (*OR* < 1, coloured blue in Table 3; e.g. ‘cancer/colon cancer is a serious illness’). Regarding the other four main considerations, people were less likely to participate in CRC screening when these played a larger role in their decision (*OR* > 1, coloured green in Table 3; e.g. ‘I feel healthy’).

**Table 3:** Main considerations associated with CRC screening participation (multiple logistic regression analysis; final model using backward selection).

Considerations ^a^^,^ ^b^	OR	95% CI ^c^
I feel healthy	1.775	1.389–2.268
I have colon problems/I have had colon problems	1.805	1.398–2.331
Cancer/colon cancer is a serious illness	.561	.434 –.726
By participating in CRC screening I will avoid serious treatment	.598	.417 –.858
By participating in CRC screening I reduce my chance of dying from colon cancer	.436	.299 –.635
By participating in CRC screening I can possibly get treated for an abnormality that would never have given me problems (= unnecessary treatment)	2.519	1.910–3.322
By participating in CRC screening I get reassured	.329	.229 –.471
It is difficult to participate in CRC screening because of health problems or physical problems	2.141	1.584–2.894

^a^ Scores range from 1 (did not play a role in decision) to 5 (played a very large role in decision)

^b^ Dependent variable: CRC screening participation (dichotomous: 1 = participated, 2 = did not participate)

^c^ All considerations in this final model were significantly associated with CRC screening participation at level p < .01

After that, we examined whether the relationship between people’s promotion-orientation, prevention-orientation or focus on advantages or disadvantages–each separately–and their CRC screening participation was mediated by each of these eight main considerations separately. In other words, we examined the direct and indirect effects between these variables (see Fig 1). Table 4 shows the considerations for which significant indirect/mediation effects were found. Regarding the relationship between promotion-orientation and CRC screening participation, indirect effects were found concerning almost all considerations (except for ‘I have colon problems/I have had colon problems’). Regarding the relationship between prevention-orientation and CRC screening participation, indirect effects were also found concerning almost all considerations (except for ‘it is difficult to participate in CRC screening because of health problems or physical problems’). The results suggest that the association between people’s promotion-orientation as well as prevention-orientation and their CRC screening participation is partially mediated by most of people’s main considerations concerning CRC screening. The direction of the direct and indirect effects was the same for both promotion-orientation and prevention-orientation. Regarding the relationship between people’s focus on advantages or disadvantages and CRC screening participation, indirect effects were found concerning the following main considerations: cancer/colon cancer is a serious illness; by participating in CRC screening I will avoid serious treatment; by participating in CRC screening I reduce my chance of dying from colon cancer; by participating in CRC screening I get reassured; it is difficult to participate in CRC screening because of health problems or physical problems. The results suggest that the association between people’s focus on advantages or disadvantages and their CRC screening participation is partially mediated by these main considerations concerning CRC screening. Sex, education and birth year were not found to be confounders in the mediation analyses, except regarding the association between promotion-orientation and prevention-orientation and the consideration ‘it is difficult to participate in CRC screening because of health problems or physical problems’. Here, education was found to be a confounder and thus corrected scores are shown.

**Table 4:** Significant indirect relationships (mediation) ^1^ between [A] promotion-orientation ^a^/[B] prevention-orientation ^a^/[C] focus on advantages or disadvantages ^b^, main considerations ^2^^,^ ^c^ and CRC screening participation ^d^. (Mediation analysis using multiple logistic regression was conducted separately for promotion-orientation, prevention-orientation, focus on advantages or disadvantages and each main consideration).

**A. Relationship between promotion-orientation, main considerations and CRC screening participation**	**OR**
*1*: *Consideration ‘I feel healthy’*	
Direct effect of promotion-orientation on participation	.261**
Direct effect of consideration on participation	1.640**
Indirect effect of promotion-orientation on participation via consideration	1.142**
*2*: *Consideration ‘Cancer/colon cancer is a serious illness’*	
Direct effect of promotion-orientation on participation	.431**
Direct effect of consideration on participation	.493**
Indirect effect of promotion-orientation on participation via consideration	.690**
*3*: *Consideration ‘By participating in CRC screening I will avoid serious treatment’*	
Direct effect of promotion-orientation on participation	.401**
Direct effect of consideration on participation	.477**
Indirect effect of promotion-orientation on participation via consideration	.742**
*4*: *Consideration ‘By participating in CRC screening I reduce my chance of dying from colon cancer’*	
Direct effect of promotion-orientation on participation	.520**
Direct effect of consideration on participation	.326**
Indirect effect of promotion-orientation on participation via consideration	.573**
*5*: *Consideration ‘By participating in CRC screening I can possibly get treated for an abnormality that would never have given me problems (= unnecessary treatment)’*	
Direct effect of promotion-orientation on participation	.239**
Direct effect of consideration on participation	1.995**
Indirect effect of promotion-orientation on participation via consideration	1.246**
*6*: *Consideration ‘By participating in CRC screening I get reassured’*	
Direct effect of promotion-orientation on participation	.549**
Direct effect of consideration on participation	.286**
Indirect effect of promotion-orientation on participation via consideration	.542**
*7*: *Consideration ‘It is difficult to participate in CRC screening because of health problems or physical problems’*	
Direct effect of promotion-orientation on participation	.291**
Direct effect of consideration on participation	2.186**
Indirect effect of promotion-orientation on participation via consideration	1.022*
**B. Relationship between prevention-orientation, main considerations and CRC screening participation**	**OR**
*1*: *Consideration ‘I feel healthy’*	
Direct effect of prevention-orientation on participation	.385**
Direct effect of consideration on participation	1.488**
Indirect effect of prevention-orientation on participation via consideration	1.127**
*2*: *Consideration ‘I have colon problems/I have had colon problems’*	
Direct effect of prevention-orientation on participation	.426**
Direct effect of consideration on participation	1.294*
Indirect effect of prevention-orientation on participation via consideration	1.018*
*3*: *Consideration ‘Cancer/colon cancer is a serious illness’*	
Direct effect of prevention-orientation on participation	.650**
Direct effect of consideration on participation	.415**
Indirect effect of prevention-orientation on participation via consideration	.669**
*4*: *Consideration ‘By participating in CRC screening I will avoid serious treatment’*	
Direct effect of prevention-orientation on participation	.601**
Direct effect of consideration on participation	.407**
Indirect effect of prevention-orientation on participation via consideration	.722**
*5*: *Consideration ‘By participating in CRC screening I reduce my chance of dying from colon cancer’*	
Direct effect of prevention-orientation on participation	.760*
Direct effect of consideration on participation	.274**
Indirect effect of prevention-orientation on participation via consideration	.571**
*6*: *Consideration ‘By participating in CRC screening I can possibly get treated for an abnormality that would never have given me problems (= unnecessary treatment)’*	
Direct effect of prevention-orientation on participation	.324**
Direct effect of consideration on participation	1.914**
Indirect effect of prevention-orientation on participation via consideration	1.340**
*7*: *Consideration ‘By participating in CRC screening I get reassured’*	
Direct effect of prevention-orientation on participation	.793*
Direct effect of consideration on participation	.227**
Indirect effect of prevention-orientation on participation via consideration	.548**
**C. Relationship between focus on advantages or disadvantages, main considerations and CRC screening participation**	**OR**
*1*: *Consideration ‘Cancer/colon cancer is a serious illness’*	
Direct effect of focus on advantages/disadvantages on participation	48.237**
Direct effect of consideration on participation	.429**
Indirect effect of focus on advantages/disadvantages on participation via consideration	1.726**
*2*: *Consideration ‘By participating in CRC screening I will avoid serious treatment’*	
Direct effect of focus on advantages/disadvantages on participation	51.581**
Direct effect of consideration on participation	.431**
Indirect effect of focus on advantages/disadvantages on participation via consideration	1.614**
*3*: *Consideration ‘By participating in CRC screening I reduce my chance of dying from colon cancer’*	
Direct effect of focus on advantages/disadvantages on participation	40.200**
Direct effect of consideration on participation	.316**
Indirect effect of focus on advantages/disadvantages on participation via consideration	2.071**
*4*: *Consideration ‘By participating in CRC screening I get reassured’*	
Direct effect of focus on advantages/disadvantages on participation	34.924**
Direct effect of consideration on participation	.296**
Indirect effect of focus on advantages/disadvantages on participation via consideration	2.384**
*5*: *Consideration ‘It is difficult to participate in CRC screening because of health problems or physical problems’*	
Direct effect of focus on advantages/disadvantages on participation	81.804**
Direct effect of consideration on participation	1.867**
Indirect effect of focus on advantages/disadvantages on participation via consideration	1.018**

^1^ See also Fig 1. ‘Direct effect of promotion-orientation/prevention orientation/focus on advantages or disadvantages on participation’ = C’-path. ‘Direct effect of consideration on participation’ = B-path. ‘Indirect effect’ = path AxB/C-C’

^2^ See Table 3 for the initial main considerations regarding CRC screening participation. Table 4 only shows those considerations involved in a significant indirect effect

^a^ Scores range from 1 (low promotion-/prevention-orientation) to 5 (high promotion-/prevention-orientation)

^b^ Dichotomous variable: score 1 = focus on advantages of CRC screening, score 2 = focus on disadvantages of CRC screening

^c^ Scores range from 1 (did not play a role in decision) to 5 (played a very large role in decision)

^d^ Dependent variable: CRC screening participation. Dichotomous variable: score 1 = participated in screening, score 2 = did not participate in screening)

* Significant at level p < .05

** Significant at level p < .001

## Discussion

Generally, CRC screening participants scored higher than non-participants on both promotion-orientation and prevention-orientation. CRC screening participants did not have a dominant goal-orientation. Although CRC screening non-participants did score significantly higher on having a prevention-orientation, their relatively low scores on both promotion- and prevention-orientation indicate they have no dominant goal-orientation as well. Regarding people’s focus on advantages or disadvantages of CRC screening, CRC screening participants in particular showed a dominant focus, namely a focus on the advantages. In addition, mediation analysis suggests support for the premise that the relationship between people’s goal-orientation or focus on the advantages or disadvantages and their CRC screening participation is partially mediated by people’s main considerations concerning CRC screening (Fig 1, Model II). Regarding differences associated with people’s sex, birth year and education, we mainly found that higher educated people were more likely not to participate in screening and that they scored lower on both promotion-orientation and prevention-orientation.

The different findings regarding the goal-orientation and focus on advantages or disadvantages of CRC screening participants and non-participants could be indicative of a different frame of mind between CRC screening participants and non-participants. It appears that CRC screening participation is associated with a focus on mainly the advantages of CRC screening and within that, advantages related to both promotion-oriented goals and prevention-oriented goals are being considered (e.g. achieving a state of being healthy as well as avoiding getting ill). This focus on the advantages could lead to CRC screening participants not noticing or valuing information about the disadvantages of CRC screening [8, 11, 16, 17], impeding the making of an informed decision [22–25]. Possibly framing information about the disadvantages in a more promotion- or prevention-oriented manner in order to make CRC screening participants notice it better is not likely to make a difference as CRC screening participants respond to both orientations equally strong. In the case of CRC screening participants being not aware enough of the possible harms of CRC screening, further research would be needed to assess what kind of orientation or framing would be effective in improving this. Regarding CRC screening non-participation, it appears there is no association with any clear frame of mind when deciding about CRC screening participation, or that we have not yet managed to capture this. The lack of a specific orientation or focus might suggest that CRC screening non-participants approach this decision open-mindedly. This then could result in a good awareness of both the possible benefits and harms of CRC screening and the making of an informed decision. However, it could also be that all information is noticed and valued less as we are not providing it in a manner that appeals to the, yet unknown, orientation or focus of CRC screening non-participants. In this case, again further research would be needed to assess what kind of orientation or framing would be effective in improving this. Additionally, we found that for CRC screening participants several of the presented considerations played a role in their decision (see S2 Appendix), whereas for non-participants the presented considerations hardly played a role in their decision, even those commonly associated with non-participation (e.g. ‘I feel healthy’ or ‘by participating I get anxious or worried’). This also suggests that, in general, achieving a positive end-state and avoiding a negative end-state regarding CRC screening is less important to CRC screening non-participants than it is to participants. Several studies have shown that often CRC screening non-participants do not view CRC screening as important or personally relevant [18, 38, 39]. Perhaps this also results in them not necessarily finding the outcome of the decision in itself, and the commonly associated considerations, as highly important or relevant. Further research is needed to shed more light on this aspect. Additionally, future research should focus on examining what the existence of different goals and frames of mind among the eligible CRC screening population in reality means for developing materials to adequately inform and support people in making their screening decision.

Our findings indicate an association between people’s goal-orientation or focus on advantages or disadvantages of CRC screening and their decision about participation. Additionally, mediation analysis suggests support for the premise that the relationship between people’s goal-orientation or focus on the advantages or disadvantages and their CRC screening participation is partially mediated by people’s main considerations concerning CRC screening. Generally, when scoring higher on both promotion-orientation and prevention-orientation and being focused on the advantages of CRC screening, those considerations that are often associated with CRC screening participation (e.g. ‘cancer is a serious illness’ or ‘by participating in CRC screening I will avoid serious treatment’) were seen as more important for people’s decision to participate. Those variables often associated with CRC screening non-participation (e.g. ‘I feel healthy’ or ‘possibility of unnecessary treatment’) were in that case either not affected or seen as less important for people’s decision to participate. These findings are as to be expected, as scoring higher on both promotion-orientation and prevention-orientation and having a focus on the advantages of CRC screening were found to be associated with CRC screening participation. However, although we conducted the mediation analysis using the commonly used method described by Baron and Kenny (1986) [36, 37], this method has been criticised and comes with several limitations [40, 41]. First, it should be recognised that we have examined possible correlations not causations. Second, based on existing literature we theorised that people’s goal-orientation or their focus on advantages or disadvantages could be affecting their CRC screening decision. Furthermore, we theorised that people’s CRC screening decision could also be affected because people’s goal-orientation or focus on advantages or disadvantages affected which considerations they have and value most in making this decision [6, 13–15]. However, although less likely based on existing literature, other possible directions could also exist, with for example, people’s considerations affecting their goal-orientation. Thus, we have examined possible mediation using correlations within a confirmatory design, resulting in our findings merely suggesting plausibility for our proposed relation. Third, we used the Sobel test to assess whether the mediation effects were significant. However, in small samples, such as our sample of CRC screening non-participants (N = 140), the Sobel test has proven not to be very potent. Therefore, results should be interpreted with some caution. Fourth, Pardo and Román (2013) [40] demonstrated that “small variations in the data (variations that are perfectly acceptable due to random sampling) can change a mediation conclusion into a non-mediation one, and the other way”. Therefore, they question the capacity to which the method described by Baron and Kenny can be used to draw valid mediation inferences.

In addition to the limitations regarding mediation analysis, our study has several other limitations. First, as we conducted a cross-sectional study where people had already decided about CRC screening participation or non-participation, we cannot be sure that this decision/behaviour in itself has not influenced their answers concerning their considerations and goal-orientation [42]. Second, we used a random sample of members of a national internet panel as participants. People who participate in online research may differ in significant ways from people who do not participate in online research, which could limit generalizability. Third, during the invitation process we mentioned that the study concerned CRC screening and people’s views on it. This could have attracted respondents who were already positive about CRC screening. On the other hand, it could also have provided an opportunity for those who were less positive about CRC screening to voice their opinion. Fourth, a relatively small proportion of our sample did not participate in CRC screening. A larger proportion might provide stronger conclusions. Furthermore, this could have an effect on the generalizability of our findings to the target population, as in 2015 27% of screening invitees chose not to participate [43]. This is a significant larger proportion than the 11% of screening non-participants in our sample. It is possible that we have examined a particular subset of non-participants that is not entirely representative of the non-participants in the population. Generalizability could also be limited by the fact that higher educated people were overrepresented in our sample. Another limitation might be that when measuring promotion-orientation and prevention-orientation we used partly different operationalisations for CRC screening participants and non-participants. Although developed as such because relevant promotion-oriented and prevention-oriented goals in the context of CRC screening participation seem to partly differ between the two groups, we might not entirely be measuring the same construct. Additionally, although the Cronbach’s alpha of .67 regarding the prevention-orientation scale for CRC screening non-participants is generally seen as acceptable, a higher value (at least above .70) would be preferred for drawing stronger conclusions. Furthermore, when assessing promotion-orientation and prevention-orientation we realistically could present CRC screening non-participants with only one statement to assess their promotion-orientation, which could be limiting the reliability. However, single-item measures have been shown to sometimes have the same predictive validity as multi-item measures [44, 45]. A strength of our study is that we examined people’s promotion-orientation and prevention-orientation as well as their focus on advantages and disadvantages of CRC screening, hereby taking different points of view regarding people’s goals surrounding their decision about CRC screening participation.

## Conclusion

Regarding CRC screening participation, CRC screening participants and non-participants differed in their goal-orientation and focus on advantages or disadvantages. CRC screening participation appears to be associated with a focus on the advantages of CRC screening and within that, both a promotion-orientation and prevention-orientation exist. This focus on the advantages could impede the making of an informed decision. CRC screening non-participation appears not to be associated with any clear goal-orientation or focus, or we have not yet managed to capture this. The lack of a specific orientation or focus might be beneficial for making an informed decision, but could also be problematic, as the current provision of information about CRC screening might not be appealing to CRC screening non-participants.

## Supporting information

S1 Appendix Questionnaire (in English and Dutch). (DOCX)

S2 Appendix Considerations regarding CRC screening participation (descriptive statistics). (DOCX)

S3 Appendix Factor analysis and correlation matrix. (DOCX)

S4 Appendix Associations between sex, education and birth year, people’s goal-orientation, people’s focus on advantages or disadvantages, and CRC screening participation and (multiple linear and logistic regression analyses). (DOCX)

## References

[1] Bergvik S, Sorlie T, Wynn R. Approach and avoidance coping and regulatory focus in patients having coronary artery bypass graft surgery. J Health Psychol. 2010;15(6):915–24. Epub 2010/05/11. 10.1177/1359105309359542 . 20453051

[2] Fuglestad PT, Rothman AJ, Jeffery RW, Sherwood NE. Regulatory Focus, Proximity to Goal Weight, and Weight Loss Maintenance. Am J Health Behav. 2015;39(5):709–20. Epub 2015/08/08. 10.5993/AJHB.39.5.12 26248180 PMC4669580

[3] Leder S, Florack A, Keller J. Self-regulation and protective health behaviour: how regulatory focus and anticipated regret are related to vaccination decisions. Psychol Health. 2015;30(2):165–88. Epub 2014/08/20. 10.1080/08870446.2014.954574 . 25137215

[4] Pfattheicher S, Sassenrath C. A regulatory focus perspective on eating behavior: how prevention and promotion focus relates to emotional, external, and restrained eating. Front Psychol. 2014;5:1314 Epub 2014/12/06. 10.3389/fpsyg.2014.01314 25477840 PMC4238324

[5] Avraham R, Van Dijk D, Simon-Tuval T. Regulatory focus and adherence to self-care behaviors among adults with type 2 diabetes. Psychol Health Med. 2016;21(6):696–706. Epub 2015/11/19. 10.1080/13548506.2015.1112413 . 26576471

[6] Eccles JS, Wigfield A. Motivational beliefs, values, and goals. Annual review of psychology. 2002;53:109–32. Epub 2001/12/26. 10.1146/annurev.psych.53.100901.135153 . 11752481

[7] Weber EU, Johnson EJ. Mindful judgment and decision making. Annual review of psychology. 2009;60:53–85. Epub 2008/09/19. 10.1146/annurev.psych.60.110707.163633 . 18798706

[8] Higgins ET. Beyond pleasure and pain. Am Psychol. 1997;52(12):1280–300. Epub 1998/01/01. . 9414606 10.1037//0003-066x.52.12.1280

[9] Summerville A, Roese NJ. Self-Report Measures of Individual Differences in Regulatory Focus: A Cautionary Note. J Res Pers. 2008;42(1):247–54. Epub 2008/05/23. 10.1016/j.jrp.2007.05.005 18496599 PMC2390858

[10] Brodscholl JC, Kober H, Higgins ET. Strategies of self‐regulation in goal attainment versus goal maintenance. European Journal of Social Psychology. 2007;37(4):628–48. 10.1002/ejsp.380

[11] Higgins ET. Making a good decision: value from fit. Am Psychol. 2000;55(11):1217–30. Epub 2001/04/03. . 11280936

[12] Allport FH. Theories of Perception and the Concept of Structure. New York: Wiley; 1955.

[13] Kunda Z. Social cognition: Making sense of people: MIT Press; 1999.

[14] Miller SM, Shoda Y, Hurley K. Applying cognitive-social theory to health-protective behavior: breast self-examination in cancer screening. Psychol Bull. 1996;119(1):70–94. Epub 1996/01/01. . 8559860 10.1037/0033-2909.119.1.70

[15] Higgins ET. How self-regulation creates distinct values: The case of promotion and prevention decision making. Journal of Consumer Psychology and Health. 2002;12:177–91.

[16] Kim Y-J. The role of regulatory focus in message framing in antismoking advertisements for adolescents. Journal of Advertising. 2006;35(1):143–51. 10.2753/JOA0091-3367350109

[17] Lee AY, Aaker JL. Bringing the frame into focus: the influence of regulatory fit on processing fluency and persuasion. J Pers Soc Psychol. 2004;86(2):205–18. Epub 2004/02/11. 10.1037/0022-3514.86.2.205 . 14769079

[18] Beydoun HA, Beydoun MA. Predictors of colorectal cancer screening behaviors among average-risk older adults in the United States. Cancer Causes Control. 2008;19(4):339–59. Epub 2007/12/19. 10.1007/s10552-007-9100-y . 18085415

[19] McCaffery K, Wardle J, Waller J. Knowledge, attitudes, and behavioral intentions in relation to the early detection of colorectal cancer in the United Kingdom. Prev Med. 2003;36(5):525–35. Epub 2003/04/12. . 12689797 10.1016/s0091-7435(03)00016-1

[20] Smith-McLallen A, Fishbein M. Predictors of intentions to perform six cancer-related behaviours: Roles for injunctive and descriptive norms. Psychology, Health & Medicine 2008;13(4):389–401. 10.1080/13548500701842933 18825578

[21] Jilcott Pitts SB, Lea CS, May CL, Stowe C, Hamill DJ, Walker KT, et al "Fault-line of an earthquake": a qualitative examination of barriers and facilitators to colorectal cancer screening in rural, Eastern North Carolina. J Rural Health. 2013;29(1):78–87. Epub 2013/01/08. 10.1111/j.1748-0361.2012.00424.x . 23289658

[22] Irwig L, McCaffery K, Salkeld G, Bossuyt P. Informed choice for screening: implications for evaluation. Bmj. 2006;332(7550):1148–50. Epub 2006/05/13. 10.1136/bmj.332.7550.1148 ; PubMed Central PMCID: PMCPmc1459621. 16690676 PMC1459621

[23] O'Connor A, O'Brien Pallas LL. Decisional conflict In: Mcfarlane GK, Mcfarlane EA, editors. Nursing diagnosis and intervention. Toronto: Mosby; 1989 p. 486–96.

[24] Marteau TM, Dormandy E, Michie S. A measure of informed choice. Health Expectations. 2001;4(2):99–108. 10.1046/j.1369-6513.2001.00140.x 11359540 PMC5060053

[25] Johansson M, Brodersen J. Informed choice in screening needs more than information. The Lancet. 2015;385(9978):1597–9. 10.1016/S0140-6736(15)60258-6 25701272

[26] Rimer BK, Briss PA, Zeller PK, Chan EC, Woolf SH. Informed decision making: What is its role in cancer screening? Cancer. 2004;101(5 Suppl):1214–28. Epub 2004/08/19. 10.1002/cncr.20512 . 15316908

[27] Jepson RG, Hewison J, Thompson A, Weller D. Patient perspectives on information and choice in cancer screening: a qualitative study in the UK. Soc Sci Med. 2007;65(5):890–9. Epub 2007/05/18. 10.1016/j.socscimed.2007.04.009 . 17507131

[28] Smith SK, Simpson JM, Trevena LJ, McCaffery KJ. Factors Associated with Informed Decisions and Participation in Bowel Cancer Screening among Adults with Lower Education and Literacy. Medical Decision Making. 2014;34(6):756–72. 10.1177/0272989X13518976 24421292

[29] Peters E. Aging-related changes in decision making In: Drolet A, Schwarz N, Yoon C, editors. The Aging Consumer: Perspectives From Psychology and Economics. New York: Routledge; 2010.

[30] Lockwood P, Jordan CH, Kunda Z. Motivation by positive and negative role models: regulatory focus determines who will best inspire us. J Pers Soc Psychol. 2002;83:854–64. 10.1037/0022-3514.83.4.854 12374440

[31] Hall NJ, Rubin GP, Dobson C, Weller D, Wardle J, Ritchie M, et al Attitudes and beliefs of non-participants in a population-based screening programme for colorectal cancer. Health Expectations. 2013;18:1645–57. 10.1111/hex.12157 . 24268129 PMC5060871

[32] Denters MJ, Deutekom M, Essink-Bot ML, Bossuyt PM, Fockens P, Dekker E. Assessing knowledge and attitudes towards screening among users of faecal immunochemical test. Health Expectations. 2013;18(5):839–49. 10.1111/hex.12056 . 23432931 PMC5060879

[33] Schwartz LM, Woloshin S, Fowler FJ, Welch HG. Enthusiasm for cancer screening in the United States. Journal of the American Medical Association. 2004;291(1):71–8. 10.1001/jama.291.1.71 14709578

[34] Hoffman RM, Elmore JG, Pignone MP, Gerstein BS, Levin CA, Fairfield KM. Knowledge and values for cancer screening decisions: Results from a national survey. Patient education and counseling. 2016;99(4):624–30. 10.1016/j.pec.2015.11.001 26603446

[35] Twisk JWR. Inleiding in de toegepaste biostatistiek [Introduction into applied biostatistics] 4 ed: Bohn Stafleu van Loghum; 2017.

[36] Baron RM, Kenny DA. The moderator–mediator variable distinction in social psychological research: Conceptual, strategic, and statistical considerations. Journal of Personality and Social Psychology. 1986;51(6):1173–82. 10.1037/0022-3514.51.6.1173 3806354

[37] MacKinnon DP, Fairchild AJ, Fritz MS. Mediation analysis. Annual review of psychology. 2007;58:593–614. Epub 2006/09/14. 10.1146/annurev.psych.58.110405.085542 16968208 PMC2819368

[38] Wools A, Dapper EA, de Leeuw JR. Colorectal cancer screening participation: a systematic review. Eur J Public Health. 2016;26(1):158–68. Epub 2015/09/16. 10.1093/eurpub/ckv148 . 26370437

[39] McCaffery K, Borril J, Williamson S, Taylor T, Sutton S, Atkin W, et al Declining the offer of flexible sigmoidoscopy screening for bowel cancer: a qualitative investigation of the decision-making process. Soc Sci Med. 2001;53(5):679–91. Epub 2001/08/02. . 11478546 10.1016/s0277-9536(00)00375-0

[40] Pardo A, Román Tabanera M. Reflections on the Baron and Kenny model of statistical mediation2013. 614–23 p.

[41] Zhao X, Lynch J, Chen Q. Reconsidering Baron and Kenny: Myths and truths about mediation analysis2010. 197–206 p.

[42] Festinger L. A Theory of Cognitive Dissonance. Stanford, CA: Stanford University Press; 1957.

[43] Leerdam MEv, Toes E, Spaander VMCM, Vuuren AJv, Dekker E, Kuipers EJ, et al. Landelijke monitoring bevolkingsonderzoek darmkanker [National monitoring colorectal cancer screening programme]—Monitor 2015—Erasmus MC / NKI-AVL. Bilthoven: Rijksinstituut voor Volksgezondheid en Milieu; 2016.

[44] Hoeppner BB, Kelly JF, Urbanoski KA, Slaymaker V. Comparative utility of a single-item versus multiple-item measure of self-efficacy in predicting relapse among young adults. J Subst Abuse Treat. 2011;41(3):305–12. Epub 2011/06/28. 10.1016/j.jsat.2011.04.005 21700411 PMC3315352

[45] Diamantopoulos A, Sarstedt M, Fuchs C, Wilczynski P, Kaiser S. Guidelines for choosing between multi-item and single-item scales for construct measurement: a predictive validity perspective. J of the Acad Mark Sci. 2012;40:434–49. 10.1007/s11747-011-0300-3

