# Supplementary material for: Do people with a different goal-orientation or specific focus make different decisions about colorectal cancer-screening participation?
Source: PLoS One. 2019 Feb 28;14(2):e0213003. doi: 10.1371/journal.pone.0213003 (PMC6394955; doi:10.1371/journal.pone.0213003)
Supplement: S1 Appendix — (DOCX) [file pone.0213003.s001.docx]

**S1. Appendix. Questionnaire (in English and Dutch)**

**A. English version**

**Participating/Not participating [adjusted accordingly] in the colorectal cancer screening programme**

In this section we will ask you about what has played a role for you in deciding that you wanted to participate/not participate [adjusted accordingly] in the colorectal cancer screening programme. Please read the questions carefully.

Question 12

We will show you a broad overview of aspects that may have played a role in your decision to participate/not participate [adjusted accordingly] in the CRC screening programme. Please indicate for every aspect to what extent it has played a role in your decision. There are no right or wrong answers. It is about what has been important *for you* in deciding that you wanted to participate/not wanted to participate [adjusted accordingly].

Three times you will be presented a segment covering different aspects. There could be aspects of which you think they are not applicable to you. We want to ask you to fill in an answer anyway. You can indicate your answer on a scale of 1 to 5. Here 1 means ‘did not play a role’ and 5 means ‘played a very large role’.

|  | **Did not play a role**  **1** | **Played a small role**  **2** | **Played a role**  **3** | **Played a large role**  **4** | **Played a very large role**  **5** |
| --- | --- | --- | --- | --- | --- |
| **Segment 1:**  **Colon problems & colon cancer** |  |  |  |  |  |
| - I feel healthy |  |  |  |  |  |
| - I have colon problems/I have had colon problems |  |  |  |  |  |
| - People I know have experience with colon |  |  |  |  |  |
| - Cancer/colon cancer is a serious illness |  |  |  |  |  |
| - I think my chance of getting colon cancer is small |  |  |  |  |  |
| - I think my chance of getting colon cancer is large |  |  |  |  |  |
| - Colon cancer is well-preventable |  |  |  |  |  |
| - Colon cancer is virtually impossible to prevent |  |  |  |  |  |
| - You can have colon cancer without having symptoms |  |  |  |  |  |
| - You will notice yourself in time if you have symptoms of colon cancer |  |  |  |  |  |
| **Segment 2:**  **Colorectal cancer screening programme** |  |  |  |  |  |
| - By participating in CRC screening I will avoid serious treatment |  |  |  |  |  |
| - By participating in CRC screening I reduce my chance of dying from colon cancer |  |  |  |  |  |
| - By participating in CRC screening I can possibly get treated for an normality that would never have given me problems (= unnecessary treatment) |  |  |  |  |  |
| - By participating in CRC screening I get reassured |  |  |  |  |  |
| - By participating in CRC screening I get anxious or worried |  |  |  |  |  |
| - The stool test^i^ does *not* give me 100% certainty about *whether* I have colon cancer |  |  |  |  |  |
| - Downsides and risks are associated with the possible follow-up test^i^ (colonoscopy) |  |  |  |  |  |
| - I think that screening programmes are generally good to participate in |  |  |  |  |  |
| - I think that screening programmes are generally *not* good to participate in |  |  |  |  |  |
| **Segment 3:**  **Social environment** |  |  |  |  |  |
| - I think most people in *my environment are positive* about CRC screening |  |  |  |  |  |
| - I think most people in *my environment are negative* about CRC screening |  |  |  |  |  |
| - I think most people in *the Netherlands are positive* about CRC screening |  |  |  |  |  |
| - I think most people *in the Netherlands are negative* about CRC screening |  |  |  |  |  |
| - I think the media mostly speak *positively* about CRC screening |  |  |  |  |  |
| - I think the media mostly speak *negatively* about CRC screening |  |  |  |  |  |
| **Other** |  |  |  |  |  |
| - It is difficult to find a suitable time to perform the stool test |  |  |  |  |  |
| - My toilet is not suitable to perform the stool test |  |  |  |  |  |
| - It is difficult to participate in CRC screening because of health problems or physical problems |  |  |  |  |  |

^i^ Stool test: The colorectal cancer screening involves a self-test. This a stool test. With the invitation you receive a small tube with a stick inside. With the stick you poke into your stool. Then you put the stick back in the small tube. You send this tube back using the return envelope. Your stool will then be examined in a laboratory.

^i^ Follow-up test/colonoscopy: When traces of blood are found in your stool using the stool test you will be referred to the hospital for follow-up testing. This follow-up test is a colonoscopy. It involves examining the inside of your colon to see if there are any indications for cancer or other abnormalities.

Question 13

Indicate which of the following two options played the largest role for you to participate/not participate [adjusted accordingly] in CRC screening. Choose the answer that fits you best.

- I mostly looked at what the advantages of CRC screening could be for me
- I mostly looked at what the disadvantages of CRC screening could be for me

Question 14 [for CRC screening participants]

You will now be presented with several options. Indicate on a scale of 1 to 5 to what extent these played a role *for you* to participate in colorectal cancer screening.

|  | **Did not play a role**  **1** | **Played a small role**  **2** | **Played a role**  **3** | **Played a large role**  **4** | **Played a very large role**  **5** |
| --- | --- | --- | --- | --- | --- |
| - I want to know whether I am healthy |  |  |  |  |  |
| - I want to maintain a good health |  |  |  |  |  |
| - I want to avoid getting ill/seriously ill |  |  |  |  |  |
| - I want to avoid risks |  |  |  |  |  |
| - I want to be reassured |  |  |  |  |  |
| - I want to prevent anxiety/worry |  |  |  |  |  |

Question 14 [for CRC screening non-participants]

You will now be presented with several options. Indicate on a scale of 1 to 5 to what extent these played a role *for you* not to participate in colorectal cancer screening.

|  | **Did not play a role**  **1** | **Played a small role**  **2** | **Played a role**  **3** | **Played a large role**  **4** | **Played a large role**  **5** |
| --- | --- | --- | --- | --- | --- |
| - I want to maintain a good health |  |  |  |  |  |
| - I want to avoid unnecessary testing or treatment |  |  |  |  |  |
| - I want to avoid risks |  |  |  |  |  |
| - I want to prevent anxiety/worry |  |  |  |  |  |

**B. Dutch version (original language of the questionnaire)**

**Meedoen/Niet meedoen [afgestemd op wat passend is] aan het bevolkingsonderzoek darmkanker**

In dit onderdeel vragen wij naar wat er voor u heeft meegespeeld bij het bepalen dat u mee ging doen/niet mee ging doen [afgestemd op wat passend is] aan het bevolkingsonderzoek darmkanker. Leest u de uitleg bij de vragen alstublieft goed door.

Vraag 12

Wij laten u een uitgebreid overzicht aan punten zien die misschien voor u hebben meegespeeld bij het bepalen dat u mee ging doen/niet mee ging doen [afgestemd op wat passend is] aan het bevolkingsonderzoek darmkanker. Wilt u per punt aankruisen in hoeverre deze voor u heeft meegespeeld? Er zijn geen goede of foute antwoorden. Het gaat om wat *voor u* belangrijk is geweest bij het bepalen dat u mee ging doen/niet mee ging doen [afgestemd op wat passend is].

U krijgt drie keer een blok met verschillende punten te zien. Het kan zijn dat er punten tussen staan waarvan u denkt dat ze niet op u van toepassing zijn. Wij willen u vragen hier toch een antwoord in te vullen. U kunt uw antwoord aangeven op een schaal van 1 tot 5. Hierbij betekent: 1 = ‘heeft niet meegespeeld’ en 5 = ‘heeft heel veel meegespeeld’.

|  | **Heeft niet meegespeeld**  **1** | **Heeft een beetje meegespeeld**  **2** | **Heeft meegespeeld**  **3** | **Heeft veel meegespeeld**  **4** | **Heeft heel veel meegespeeld**  **5** |
| --- | --- | --- | --- | --- | --- |
| **Blok 1:**  **Darmklachten & darmkanker** |  |  |  |  |  |
| - Ik voel mijzelf gezond |  |  |  |  |  |
| - Ik heb darmklachten (gehad) |  |  |  |  |  |
| - Mensen in mijn omgeving hebben ervaring met darmklachten of darmkanker |  |  |  |  |  |
| - (Darm)kanker is een ernstige ziekte |  |  |  |  |  |
| - Mijn kans op het krijgen van darmkanker is denk ik (vrij) klein |  |  |  |  |  |
| - Mijn kans op het krijgen van darmkanker is denk ik (vrij) groot |  |  |  |  |  |
| - Darmkanker is goed te voorkomen |  |  |  |  |  |
| - Darmkanker is (bijna) niet te voorkomen |  |  |  |  |  |
| - Je kunt (ernstige) darmkanker hebben zonder dat je klachten hebt |  |  |  |  |  |
| - Je merkt bij darmkanker zelf op tijd wanneer je echt problemen of klachten hebt |  |  |  |  |  |
| **Blok 2:**  **Bevolkingsonderzoek darmkanker** |  |  |  |  |  |
| - Door meedoen aan het bevolkingsonderzoek darmkanker voorkom ik een ingrijpende behandeling |  |  |  |  |  |
|  |  |  |  |  |  |
| - Door meedoen aan het bevolkingsonderzoek darmkanker verklein ik mijn kans op het overlijden aan darmkanker |  |  |  |  |  |
| - Door meedoen aan het bevolkingsonderzoek darmkanker word ik mogelijk behandeld voor een afwijking waar ik nooit last van zou hebben gekregen (= onnodige behandeling) |  |  |  |  |  |
| - Door meedoen aan het bevolkingsonderzoek darmkanker word ik gerustgesteld |  |  |  |  |  |
| - Door meedoen aan het bevolkingsonderzoek darmkanker word ik ongerust of bang gemaakt |  |  |  |  |  |
| - De ontlastingstest^i^ geeft mij *geen* 100% zekerheid geeft over het *wel of niet* hebben van darmkanker |  |  |  |  |  |
| - Er zitten nadelen en risico’s aan het doen van het eventuele vervolgonderzoek^i^ (een coloscopie) |  |  |  |  |  |
| - Ik vind bevolkingsonderzoeken meestal goed om aan mee te doen |  |  |  |  |  |
| - Ik vind bevolkingsonderzoeken meestal *niet* goed om aan mee te doen |  |  |  |  |  |
| **Blok 3:**  **Omgeving** |  |  |  |  |  |
| - Ik denk dat de meeste mensen in *mijn omgeving* *positief* zijn over het bevolkingsonderzoek darmkanker |  |  |  |  |  |
|  |  |  |  |  |  |
| - Ik denk dat de meeste mensen in *mijn omgeving* *negatief* zijn over het bevolkingsonderzoek |  |  |  |  |  |
| - Ik denk dat de meeste mensen in *Nederland* *positief* zijn over het bevolkingsonderzoek darmkanker |  |  |  |  |  |
| - Ik denk dat de meeste mensen in *Nederland* *negatief* zijn over het bevolkingsonderzoek darmkanker |  |  |  |  |  |
| - Ik denk dat in de media vooral *positief* wordt gesproken over het bevolkingsonderzoek darmkanker |  |  |  |  |  |
| - Ik denk dat in de media vooral *negatief* wordt gesproken over het bevolkingsonderzoek darmkanker |  |  |  |  |  |
| **Overig** |  |  |  |  |  |
| - Het is lastig om een geschikt moment te vinden om de ontlastingstest te doen |  |  |  |  |  |
|  |  |  |  |  |  |
| - Mijn toilet is niet (goed) geschikt om de ontlastingstest te doen |  |  |  |  |  |
| - Het is lastig om mee te doen aan het bevolkingsonderzoek darmkanker door gezondheidsproblemen of fysieke problemen |  |  |  |  |  |

^i^ Ontlastingstest: Het bevolkingsonderzoek bestaat uit een zelfafnametest. Dit is een ontlastingstest. Bij de uitnodiging ontvangt u een buisje met daarin een staafje. Met het staafje prikt u in uw ontlasting. Het staafje doet u vervolgens terug in het buisje. Dit buisje stuurt u terug in de retourenvelop. Uw ontlasting wordt daarna in een laboratorium onderzocht.

^i^ Vervolgonderzoek/coloscopie: Wanneer er via de ontlastingstest bloed in de ontlasting wordt gevonden, wordt u doorgestuurd naar het ziekenhuis om vervolgonderzoek te doen. Dit vervolgonderzoek is een coloscopie. Hierbij wordt de binnenkant van de darmen onderzocht om te zien of er aanwijzingen zijn voor kanker of andere afwijkingen.

Vraag 13

Geef aan welke van de volgende twee antwoordmogelijkheden *voor u* vooral heeft meegespeeld om mee te doen/niet mee te doen [afgestemd op wat passend is] aan het bevolkingsonderzoek darmkanker. Kruis het antwoord aan dat het best bij u past.

- Ik heb vooral gekeken naar wat voor mij de voordelen kunnen zijn van het bevolkingsonderzoek
- Ik heb vooral gekeken naar wat voor mij de nadelen kunnen zijn van het bevolkingsonderzoek

Vraag 14 [voor WEL deelnemers]

U krijgt nu een aantal opties te zien. Geef voor elk op een schaal van 1 tot 5 aan in hoeverre deze *voor u* heeft meegespeeld om mee te doen aan het bevolkingsonderzoek darmkanker.

|  | **Heeft niet mee-gespeeld**  **1** | **Heeft een beetje mee-gespeeld**  **2** | **Heeft mee-**  **gespeeld**  **3** | **Heeft veel mee-**  **gespeeld**  **4** | **Heeft voor mij heel veel mee-gespeeld**  **5** |
| --- | --- | --- | --- | --- | --- |
| - Ik wil weten of ik gezond ben |  |  |  |  |  |
| - Ik wil een goede gezondheid behouden |  |  |  |  |  |
| - Ik wil (ernstig) ziek worden voorkomen |  |  |  |  |  |
| - Ik wil risico’s vermijden |  |  |  |  |  |
| - Ik wil gerustgesteld worden |  |  |  |  |  |
| - Ik wil onrust/zorgen voorkomen |  |  |  |  |  |

Vraag 14 [voor NIET deelnemers]

U krijgt nu een aantal opties te zien. Geef voor elk op een schaal van 1 tot 5 aan in hoeverre deze *voor u* heeft meegespeeld om niet mee te doen aan het bevolkingsonderzoek darmkanker.

|  | **Heeft niet mee-gespeeld**  **1** | **Heeft een beetje mee-gespeeld**  **2** | **Heeft mee-gespeeld**  **3** | **Heeft veel mee-gespeeld**  **4** | **Heeft voor mij heel veel mee-gespeeld**  **5** |
| --- | --- | --- | --- | --- | --- |
| - Ik wil een goede gezondheid behouden |  |  |  |  |  |
| - Ik wil onnodig onderzoek of behandeling voorkomen |  |  |  |  |  |
| - Ik wil risico’s vermijden |  |  |  |  |  |
| - Ik wil onrust/zorgen voorkomen |  |  |  |  |  |
