# Supplementary material for: Do people with a different goal-orientation or specific focus make different decisions about colorectal cancer-screening participation?
Source: PLoS One. 2019 Feb 28;14(2):e0213003. doi: 10.1371/journal.pone.0213003 (PMC6394955; doi:10.1371/journal.pone.0213003)
Supplement: S2 Appendix — (DOCX) [file pone.0213003.s002.docx]

**S2. Appendix. Considerations regarding CRC screening participation (descriptive statistics)**

| **Considerations ^a^** | ***Total sample ^1^*** | ***CRC screening participants ^2^*** | ***CRC screening non-participants ^3^*** |
| --- | --- | --- | --- |
|  | **M (SD)** | **M (SD)** | **M (SD)** |
| *Theme: Colon problems & colon cancer* |  |  |  |
| I feel healthy | 2.02 (1.29) | 1.98 (1.27) | 2.34 (1.38) |
| I have colon problems/I have had colon  problems | 1.60 (1.14) | 1.57 (1.09) | 1.84 (1.48) |
| People I know have experience with colon  problems or colorectal cancer | 2.38 (1.43) | 2.45 (1.44) | 1.76 (1.23) |
| Cancer/colon cancer is a serious illness | 3.77 (1.23) | 3.95 (1.07) | 2.30 (1.40) |
| I think my chance of getting colon cancer is  small | 1.84 (1.05) | 1.82 (1.01) | 1.99 (1.31) |
| I think my chance of getting colon cancer is  large | 1.84 (1.13) | 1.87 (1.12) | 1.63 (1.15) |
| Colon cancer is well-preventable | 2.48 (1.29) | 2.57 (1.29) | 1.76 (1.10) |
| Colon cancer is virtually impossible to  prevent | 1.87 (1.11) | 1.90 (1.12) | 1.58 (.99) |
| You can have colon cancer without having  symptoms | 3.03 (1.40) | 3.16 (1.36) | 1.99 (1.25) |
| You will notice yourself in time if you have  symptoms of colon cancer | 1.63 (1.02) | 1.62 (1.02) | 1.74 (1.04) |
| *Theme: Colorectal cancer screening programme* |  |  |  |
| By participating in CRC screening I will  avoid serious treatment | 2.95 (1.37) | 3.11 (1.31) | 1.59 (1.05) |
| By participating in CRC screening I reduce  my chance of dying from colon cancer | 3.28 (1.31) | 3.49 (1.18) | 1.57 (1.05) |
| By participating in CRC screening I can  possibly get treated for an abnormality that  would never have given me problems (=  unnecessary treatment) | 2.03 (1.30) | 1.97 (1.26) | 2.50 (1.52) |
| By participating in CRC screening I get  reassured | 2.97 (1.29) | 3.16 (1.20) | 1.41 (.91) |
| By participating in CRC screening I get  anxious or worried | 1.37 (.81) | 1.31 (.71) | 1.91 (1.31) |
| The stool test does not give me 100%  certainty about whether I have colon cancer | 2.07 (1.17) | 2.07 (1.14) | 2.15 (1.33) |
| Downsides and risks are associated with the  possible follow-up test (colonoscopy) | 1.78 (1.13) | 1.70 (1.05) | 2.40 (1.54) |
| I think that screening programmes are  generally good to participate in | 3.58 (1.29) | 3.76 (1.16) | 2.09 (1.34) |
| I think that screening programmes are  generally not good to participate in | 1.23 (.75) | 1.17 (.63) | 1.74 (1.27) |
| *Theme: Social environment* |  |  |  |
| I think most people in my environment are  positive about CRC screening | 2.23 (1.36) | 2.32 (1.37) | 1.53 (.98) |
| I think most people in my environment are  negative about CRC screening | 1.16 (.56) | 1.14 (.52) | 1.34 (.83) |
| I think most people in the Netherlands are  positive about CRC screening | 2.14 (1.33) | 2.21 (1.35) | 1.55 (1.05) |
| I think most people in the Netherlands are  negative about CRC screening | 1.17 (.55) | 1.16 (.55) | 1.23 (.63) |
| I think the media mostly speak positively  about CRC screening | 2.13 (1.25) | 2.19 (1.26) | 1.60 (1.00) |
| I think the media mostly speak negatively  about CRC screening | 1.22 (.60) | 1.20 (.59) | 1.34 (.73) |
| *Theme: Other* |  |  |  |
| It is difficult to find a suitable time to  perform the stool test | 1.42 (.92) | 1.41 (.88) | 1.57 (1.19) |
| My toilet is not suitable to perform the stool  test | 1.38 (.90) | 1.36 (.87) | 1.53 (1.17) |
| It is difficult to participate in CRC screening  because of health problems or physical  problems | 1.16 (.68) | 1.11 (.53) | 1.63 (1.32) |

^1^ N = 1282

^2^ N = 1142

^3^ N = 140

^a^ Scores range from 1 (did not play a role in decision) to 5 (played a very large role in decision)
