# Supplementary material for: Do people with a different goal-orientation or specific focus make different decisions about colorectal cancer-screening participation?
Source: PLoS One. 2019 Feb 28;14(2):e0213003. doi: 10.1371/journal.pone.0213003 (PMC6394955; doi:10.1371/journal.pone.0213003)
Supplement: S3 Appendix — (DOCX) [file pone.0213003.s003.docx]

**S3. Appendix. Factor analysis and Correlation matrix**

**A. Factor analysis**

Confirmatory factor analysis (CFA; oblique/Direct oblimin rotation) was performed on the items used to measure goal-orientation and focus on the advantages or disadvantages regarding colorectal cancer (CRC) screening.

**Measurement CRC screening participants**

| **Items** | **Factor 1** | **Factor 2** |
| --- | --- | --- |
| Focus on the advantages or disadvantages ^a^ | .000 | .**999** |
| I want to know whether I am healthy ^b^ | **.823** | .024 |
| I want to maintain a good health ^b^ | **.841** | -.003 |
| I want to avoid getting ill/seriously ill ^b^ | **.820** | .020 |
| I want to avoid risks ^b^ | **.843** | -.004 |
| I want to be reassured ^b^ | **.832** | -.039 |
| I want to prevent anxiety/worry ^b^ | **.839** | -.001 |

Extraction Method: Principal Component Analysis.

Rotation Method: Oblimin with Kaiser Normalization.

Rotation converged in 2 iterations.

^a^ Intended to measure people’s focus on the advantages or disadvantages of CRC screening

^b^ Intended to measure people’s goal-orientation

**Measurement CRC screening non-participants**

| **Items** | **Factor 1** | **Factor 2** |
| --- | --- | --- |
| Focus on the advantages or disadvantages ^a^ | .093 | .**956** |
| I want to maintain a good health ^b^ | **.859** | -.003 |
| I want to avoid unnecessary testing or treatment ^b^ | **.519** | -.175 |
| I want to avoid risks ^b^ | **.845** | .119 |
| I want to prevent anxiety/worry ^b^ | **.664** | .263 |

Extraction Method: Principal Component Analysis.

Rotation Method: Oblimin with Kaiser Normalization.

Rotation converged in 4 iterations.

^a^ Intended to measure people’s focus on the advantages or disadvantages of CRC screening

^b^ Intended to measure people’s goal-orientation

**B. Correlation matrix**

Correlations between the items used to assess people’s goal-orientation, focus on advantages or disadvantages, and their ‘main considerations’ regarding CRC screening.

**Measurement CRC screening participants**

|  | **Focus / goal-** | **orientation item** |  |  |  |  |  |
| --- | --- | --- | --- | --- | --- | --- | --- |
| **Main**  **consideration**  **item** | *Focus on the advantages/*  *disadvantages ^a^* | *I want to know whether I am healthy ^b^* | *I want to maintain a good health ^b^* | *I want to avoid getting ill/seriously ill ^b^* | *I want to avoid risks ^b^* | *I want to be reassured ^b^* | *I want to prevent anxiety/worry ^b^* |
| *I feel healthy* | .013 | .203** | .208** | .158** | .171** | .208** | .207** |
| *I have colon problems/I have had colon problems* | -.037 | .123** | .093** | .145** | .113** | .130** | .123** |
| *Cancer/colon cancer is a serious illness* | -.088** | .343** | .368** | .436** | .372** | .306** | .308** |
| *By participating in CRC screening I will avoid serious treatment* | -.060* | .338** | .328** | .394** | .396** | .328** | .338** |
| *By participating in CRC screening I reduce my chance of dying from colon cancer* | -.032 | .429** | .477** | .544** | .466** | .373** | .358** |
| *By participating in CRC screening I can possibly get treated for an abnormality that would never have given me problems (= unnecessary treatment)* | .060* | .222** | .190** | .166** | .259** | .295** | .287** |
| *By participating in CRC screening I get reassured* | -.083** | .489** | .465** | .446** | .473** | .631** | .577** |
| *It is difficult to participate in CRC screening because of health problems or physical problems* | .017 | .012 | .025 | .011 | .033 | .044 | .038 |

* p < .05

** p < .001

**Measurement CRC screening non-participants**

|  | **Focus / goal-** | **orientation item** |  |  |  |
| --- | --- | --- | --- | --- | --- |
| **Main**  **consideration**  **item** | *Focus on the advantages/*  *disadvantages ^a^* | *I want to maintain a good health ^b^* | *I want to avoid unnecessary testing or treatment ^b^* | *I want to avoid risks ^b^* | *I want to prevent anxiety/worry ^b^* |
| *I feel healthy* | .176* | .236** | .344** | .113 | .159 |
| *I have colon problems/I have had colon problems* | -.319** | -.053 | -.002 | -.050 | -.178* |
| *Cancer/colon cancer is a serious illness* | -.159 | .272** | .144 | .201* | .106 |
| *By participating in CRC screening I will avoid serious treatment* | -.195* | .221** | .081 | .130 | .059 |
| *By participating in CRC screening I reduce my chance of dying from colon cancer* | -.268** | .332** | -.029 | .195* | .082 |
| *By participating in CRC screening I can possibly get treated for an abnormality that would never have given me problems (= unnecessary treatment)* | .142 | .380** | .442** | .273** | .451** |
| *By participating in CRC screening I get reassured* | -.244** | .264** | .008 | .177* | .101 |
| *It is difficult to participate in CRC screening because of health problems or physical problems* | .000 | .095 | -.097 | .124 | -.017 |

* p < .05

** p < .001
