# Supplementary material for: Do people with a different goal-orientation or specific focus make different decisions about colorectal cancer-screening participation?
Source: PLoS One. 2019 Feb 28;14(2):e0213003. doi: 10.1371/journal.pone.0213003 (PMC6394955; doi:10.1371/journal.pone.0213003)
Supplement: S4 Appendix — (DOCX) [file pone.0213003.s004.docx]

**S4. Appendix. Associations between sex, education and birth year, people’s goal-orientation, people’s focus on advantages or disadvantages, and CRC screening participation and (multiple linear and logistic regression analyses)**

| **Variable** | **B** | **95% CI** |
| --- | --- | --- |
| *Promotion-orientation ^a^* |  |  |
| Sex ^b^ | -.045 | -.178 – .089 |
| Education ^c^ |  |  |
| Intermediate | .042 | -.141 – .225 |
| High | -.174* | -.346 – -.002 |
| Birth year ^d^ |  |  |
| 1945 | .036 | -.218 – .290 |
| 1953 | .099 | -.141 – .339 |
| 1955 | .004 | -.239 – .248 |
| 1957 | .038 | -.206 – .281 |
| *Prevention-orientation ^a^* |  |  |
| Sex ^b^ | -.008 | -.135 – .118 |
| Education ^c^ |  |  |
| Intermediate | .002 | -.172 – .175 |
| High | -.195* | -.358 – -.032 |
| Birth year ^d^ |  |  |
| 1945 | .020 | -.221 – .261 |
| 1953 | .112 | -.116 – .340 |
| 1955 | .005 | -.226 – .236 |
| 1957 | .081 | -.150 – .313 |
|  | **OR** | **95% CI** |
| *Focus on advantages – Focus on disadvantages ^e^* |  |  |
| Sex ^b^ | .896 | .572 – 1.402 |
| Education ^c^ |  |  |
| Intermediate | 1.365 | .687 – 2.714 |
| High | 1.813 | .963 – 3.412 |
| Birth year ^d^ |  |  |
| 1945 | .614 | .300 – 1.255 |
| 1953 | .509 | .257 – 1.007 |
| 1955 | .648 | .332 – 1.266 |
| 1957 | .347* | .163 – .738 |
| *CRC screening participation* *^f^* |  |  |
| Sex ^b^ | 1.133 | .785 – 1.635 |
| Education ^c^ |  |  |
| Intermediate | 1.737 | .964 – 3.131 |
| High | 2.173* | 1.251 – 3.776 |
| Birth year ^d^ |  |  |
| 1945 | .626 | .324 – 1.211 |
| 1953 | .707 | .387 – 1.290 |
| 1955 | .752 | .410 – 1.380 |
| 1957 | .574 | .306 – 1.078 |

^a^ Scores range from 1 (low promotion-/prevention-orientation) to 5 (high promotion-/prevention-orientation)

^b^ Reference category is men

^c^ Reference category is low education

^d^ Reference category is year of birth 1941

^e^ Dichotomous variable: score 1= focus on advantages, score 2 = focus on disadvantages

^f^ Dichotomous variable: score 1 = participated in CRC screening, score 2 = did not participate in CRC screening

* Significant at level p < .05

** Significant at level p < .001
